# The evolution of metapopulation dynamics and the number of stem cells in intestinal crypts and other tissue structures in multicellular bodies

**DOI:** 10.1111/eva.13069

**Published:** 2020-08-12

**Authors:** David Birtwell, Georg Luebeck, Carlo C. Maley

**Affiliations:** ^1^ Norris Comprehensive Cancer Center University of Southern California Los Angeles CA USA; ^2^ Public Health Sciences Division Fred Hutchinson Cancer Research Center Seattle WA USA; ^3^ Arizona Cancer Evolution Center Biodesign Institute and School of Life Sciences Arizona State University Tempe AZ USA

**Keywords:** cancer, evolution, initiation, metapopulation dynamics, neoplastic progression, simulation

## Abstract

Carcinogenesis is a process of somatic evolution. Previous models of stem and transient amplifying cells in epithelial proliferating units like colonic crypts showed that intermediate numbers of stem cells in a crypt should optimally prevent progression to cancer. If a stem cell population is too small, it is easy for a mutator mutation to drift to fixation. If it is too large, it is easy for selection to drive cell fitness enhancing carcinogenic mutations to fixation. Here, we show that a multiscale microsimulation, that captures both within‐crypt and between‐crypt evolutionary dynamics, leads to a different conclusion. Epithelial tissues are metapopulations of crypts. We measured time to initiation of a neoplasm, implemented as inactivation of both alleles of a tumor suppressor gene. In our model, time to initiation is dependent on the spread of mutator clones in the crypts. The proportion of selectively beneficial and deleterious mutations in somatic cells is unknown and so was explored with a parameter. When the majority of non‐neutral mutations are deleterious, the fitness of mutator clones tends to decline. When crypts are maintained by few stem cells, intercrypt competition tends to remove crypts with fixed mutators. When there are many stem cells within a crypt, there is virtually no crypt turnover, but mutator clones are suppressed by within‐crypt competition. If the majority of non‐neutral mutations are beneficial to the clone, then these results are reversed and intermediate‐sized crypts provide the most protection against initiation. These results highlight the need to understand the dynamics of turnover and the mechanisms that control homeostasis, both at the level of stem cells within proliferative units and at the tissue level of competing proliferative units. Determining the distribution of fitness effects of somatic mutations will also be crucial to understanding the dynamics of tumor initiation and progression.

## 1 INTRODUCTION

The organization of a population into spatially distinct subpopulations can have a dramatic effect on the evolution of that metapopulation (Hanski & Gaggiotti, 2004). This has implications for both the evolution of organisms and for the effect of tissue architecture on somatic evolution and tissue health. In multicellular organisms, epithelia are typically divided into subpopulations of tissue stem cells along with the transient amplifying cells and differentiated cells that they produce. These subpopulations go by different names in different tissues, such as crypts in the intestine, or more generally, epithelial proliferative units. Cairns first recognized that the division of stem cells into subpopulations, such as crypts, acts as a tumor suppressor (Cairns, 1975). A mutant stem cell with a reproductive or survival advantage may take over a crypt, but is generally constrained from expanding beyond that subpopulation unless it breaches the crypt via a process known as “crypt fission” which tends to duplicate the mutant crypt cell population. However, by establishing a (population) size barrier the mutant clone has to overcome, the subpopulation structure of the tissue limits the probability that that clone will acquire further carcinogenic mutations. Yet, clones of mutant stem cells can be observed at scales spanning many crypt diameters, especially in compromised tissues such as ulcerative colitis and Barrett's esophagus (Maley et al., 2004; Salk et al., 2009). A fundamental question is therefore how the crypt‐level metapopulation dynamics affect the accumulation of somatic mutations during carcinogenesis.

Here, we explore the evolutionary dynamics of mutant stem populations that lead to tumor initiation, that is, the breach of the crypt barrier allowing clonal expansions of crypts across a tissue, as well as of mutant stem cells within (and out of) a crypt. While there may be multiple pathways to tumor initiation, it has been shown that the inactivation of a single tumor suppressor gene (TSG) such as the adenomatous polyposis coli (APC) gene in colon is sufficient to abrogate crypt homeostasis, leading to the formation of aberrant crypts and nascent adenomas (Humphries & Wright, 2008). Using an agent‐based microsimulation model for both stem cell turnover within a crypt and for the crypt‐population (tissue‐level) dynamics, we study the role of pretumor evolution in tumor initiation. This exploration allows for the selection of mutant crypts across the tissue prior to the inactivation of the tumor suppressor gene—a form of premalignant field cancerization—while the stem cell in which the tumor suppressor is inactivated can proliferate beyond the limit of a single crypt, due to crypt bifurcation.

The evolution of somatic cells is a complex multiscale process depending on the nature of somatic mutations, which may either increase or decrease cell fitness, stem cell divisions, and differentiation or apoptosis, as well as subpopulation (e.g., crypt) division and extinction rates. There is considerable evidence that carcinogenesis involves both an increase in the rate of (epi)genetic lesions (Bielas, Loeb, Rubin, True, & Loeb, 2006; Breivik, 2005; Ji & King, 2001; Weisenberger et al., 2006) and expansions of clones with a relative fitness advantage over their competitor cells (Cannataro, Gaffney, & Townsend, 2018; Maley et al., 2004; Pepper, Findlay, Kassen, Spencer, & Maley, 2009; Vermeulen et al., 2013; Williams et al., 2018). However, it continues to be unclear whether the mutator phenotype is a pre‐initiation phenomenon in carcinogenesis or is more likely to occur during tumor progression. In Barrett's esophagus, another crypt‐structured precancer, we found evidence that genomic instability precedes genome doubling and transformation (Martinez et al., 2018). The frequency of deleterious versus beneficial mutations in somatic cells is also unknown, though the large number of genes in metazoans devoted to differentiation, apoptosis, and cell cycle control suggests that the frequency of deleterious mutations may be lower in somatic evolution than organismal evolution (Rajagopalan, Nowak, Vogelstein, & Lengauer, 2003). Recent analysis of somatic mutations in cancer found no evidence of purifying selection, except in a few essential genes, and strong evidence of positive selection, with large selective effects (Williams et al., 2018), suggesting that beneficial mutations are more common than deleterious mutations in somatic evolution (Martincorena et al., 2017). Although a definitive answer to these questions can only come from further experimental data, a theoretical exploration that recognizes the roles of metapopulation dynamics, the mutator phenotype, and the proportion of deleterious to advantageous mutations in the process of tumor initiation is called for. Such an exploration will help the identification of factors that drive the tumor initiation process.

Our model integrates previous efforts to characterize the stem cell dynamics within a crypt (Cannataro, McKinley, & St Mary, 2016; Cannataro, McKinley, & St. Mary, 2017; Frank, Iwasa, & Nowak, 2003; Komarova, 2005; Komarova & Cheng, 2006; Loeffler, Birke, Winton, & Potten, 1993; Meineke, Potten, & Loeffler, 2001; Michor, Frank, May, Iwasa, & Nowak, 2003; Nowak et al., 2003; Pepper, Sprouffske, & Maley, 2007) with models of the dynamics of crypt populations (Cannataro et al., 2017; Chao, Eck, Brash, Maley, & Luebeck, 2008; Kostadinov, Maley, & Kuhner, 2016; Loeffler, Bratke, Paulus, Li, & Potten, 1997; Totafurno, Bjerknes, & Cheng, 1987). Mathematical studies of the stem cell population in the crypt niche suggest that (epi)genetic alterations that increase the rate of genetic lesions (mutator mutations) and reduce the fitness of stem cells will tend to drift to fixation if the stem cell population is small, whereas carcinogenic mutations that increase the proliferation or survival of a stem cell will tend to spread if the stem cell population is large (Cannataro et al., 2016, 2017; Komarova, 2005; Michor et al., 2003), assuming that most non‐neutral somatic mutations are deleterious. The accumulation of deleterious mutations may lead to senescence of the intestine over time (Cannataro et al., 2016). However, competition between crypts of different fitnesses may significantly change the dynamics of the establishment of a mutator clone, through a metapopulation dynamic. Our in silico experiments suggest that there may have been selection at the level of the organism to minimize the number of stem cells within each subpopulation of its structured epithelium so as to reduce the probability of tumor suppressor gene inactivation and the initiation of carcinogenesis.

## 2 Methods

We implemented a multiscale model of epithelial tissue architecture with stem cells subdivided into crypts under homeostatic control. We examined the time required until the two alleles of a tumor suppressor gene (TSG) were inactivated in at least one stem cell to represent tumor initiation. The model was run at least 50 times for every parameter setting. Crypts were arranged in a flat hexagonal tissue, similar to that observed in colon, and contained a population of stem cells as well as an implicitly modeled transient amplifying compartment. Stem cells divided both symmetrically and asymmetrically. Symmetric division resulted in two daughter stem cells each having the opportunity during the division event (synthesis) to acquire a mutation. Asymmetric division did not result in any new stem cells, but did provide an opportunity for stem cell mutation. Stem cell loss, due to cell death or differentiation, and stem cell gain due to division events were modeled as a stochastic birth–death process with parameters that were functions of the stem cell fitness and of homeostatic feedback effects in response to deviations of the crypt cell population from its normal target level (Equations 1 and 2). A flow chart of the model algorithm is shown in Figure S1.

Homeostasis operated at two spatial scales. Within a crypt, if the stem cell population dropped below the target level, stem cell division rates increased by a parameterized amount (Equation 3). If the population rose above the target level, stem cell loss rates increased (Equation 2). The level of homeostatic feedback was proportional to the degree of deviation away from the target equilibrium level (Equations 2 and 3). We also introduced a mechanism for homeostasis on the hexagonal lattice of crypts. If all the stem cells in a crypt died, the inhibition on stem cell population growth was released from the neighboring crypts. When the stem cell population of a neighbor reached twice the equilibrium level, we modeled crypt bifurcation by allocating half of its stem cells to a new crypt in the location of the dead neighbor.

We included both beneficial mutations that increased the division probability or the survival probability of stem cells as well as deleterious mutations that decreased them. These can accumulate indefinitely and affect fitness multiplicatively (Equation 4). We also implemented a genetic instability mutation that increased the clone's mutation rate 100‐fold (Bielas et al., 2006; Herr, Kennedy, Knowels, Schultz, & Preston, 2014; Ji & King, 2001). The frequency of each mutation type (those that changed the cell's fitness, the proportion of non‐neutral mutations that were deleterious, as well as the rate of TSG inactivation) was set by parameters. Each mutation affected proliferation, survival, or mutation rate parameters by a constant factor. Half of the deleterious mutations decreased stem cell proliferation, and half decreased their survival (increased cell loss). For the beneficial mutations, 40% increased the cell's proliferation rate, 40% decreased stem cell loss, and 20% caused the mutator phenotype.

The following equations and assumptions govern the model

### 2.1 Equations

Equation 1: Time to stem cell loss

Let *t* be a random exponential deviate with distribution function $f_{r}\left(t\right)$ and rate parameter *r*. The time to cell loss due to apoptosis or differentiation is the minimum of the time to cell loss due to background cell death or differentiation and the time to cell loss due to crypt feedback.

(1) $$t_{\text{cell}\_\text{loss}}=\min\left(t_{1}∼f_{\text{background}\_\text{loss}},t_{2}∼f_{\text{feedback}\_\text{loss}}\right).$$

Equation 2: Homeostatic crypt feedback by differentiation

When the stem cell population within a crypt expands beyond the homeostatic target level ($k_{\text{crypt\textbackslash{}\_size}}$), the crypt provides homeostatic feedback via a change in the rate of stem cell loss with a rate parameter equal to the base stem cell loss rate multiplied by the crypt feedback multiplier. The crypt feedback rate multiplier is used to calculate the time to stem cell loss due to crypt homeostatic feedback. The crypt feedback multiplier is equal to 2 raised to the nth power where n is the excess number of stem cells above the crypt size, divided by the $k_{\text{crypt\textbackslash{}\_deviation}}$ parameter. Here, $k_{\text{crypt\textbackslash{}\_deviation}}=0.2$ and $k_{\text{crypt\textbackslash{}\_size}}$ is a parameter that we varied across experiments.

(2) $$r_{\text{feedback\textbackslash{}\_cell}\_\text{loss}}=r_{\text{base}\_\text{cell}\_\text{loss}}2^{\max\left(0,n_{\text{cells}}‐k_{\text{crypt}\_\text{size}}\right)/k_{\text{crypt}\_\text{deviation}}}$$

Equation 3: Homeostatic crypt feedback by proliferation

When the stem cell population of a crypt drops below $k_{\text{crypt\textbackslash{}\_size}}$, the division rate of the remaining stem cells is increased by a factor that depends on the difference between the current number of stem cells (*n*_cells_)and $k_{\text{crypt\textbackslash{}\_size}}$:

(3) $$r_{\text{feedback}\_\text{division}}=r_{\text{base}\_\text{division}}2^{\max\left(0,k_{\text{crypt}\_\text{size}}‐n_{\text{cells}}\right)/k_{\text{crypt}\_\text{deviation}}}$$

Equation 4: Fitness mutation effects

$k_{\text{fitness}}$ is a constant factor representing the effect of a single beneficial mutation on fitness. As a first approximation, we assume that there are many possible mutations that increase and decrease the fitness of a somatic clone by approximately the same amount, and so, the effect of n beneficial mutations ($n_{\text{beneficial}}$) on stem cell fitness is the constant fitness effect raised to the nth power. The effect of *n* deleterious mutations ($n_{\text{deleterious}}$) of small effect is just the inverse of $k_{\text{fitness}}$ raised to the nth power. There is a separate $m_{\text{fitness}}$ calculated for the division probability and the survival probability of a cell, because beneficial and deleterious mutations may affect either of those probabilities.

(4) $$m_{\text{fitness}}={\left(k_{\text{fitness}}\right)}^{n_{\text{beneficial}}}{\left(\frac{1}{k_{\text{fitness}}}\right)}^{n_{\text{deleterious}}}$$

### 2.2 Assumptions

Crypts consist of stem cells and of transient amplifying cells.

Crypt density is fixed, that is, the tissue contains a fixed number of crypts arranged on a hexagonal grid.

The number of cells in a crypt transient amplifying compartment is fixed.

Crypts attempt to maintain a stable population of stem cells through homeostatic feedback. When the number of stem cells drops below the target level, the division rate of each stem cell in the crypt is increased. When the number of stem cells grows above the target level, the cell loss rate of each stem cell in the crypt is increased.

Crypts divide to fill vacant slots left by adjacent crypts that have gone extinct due to loss of the constituent stem cells.

The extinction of an adjacent crypt suppresses the homeostatic apoptotic signals, allowing the stem cell populations in neighboring crypts to expand. Once that extinct crypt is replaced, the normal homeostatic controls on stem cell numbers of neighboring crypts are restored.

Crypt division is triggered by an expansion of the stem cell population of a crypt to twice its homeostatic level, as hypothesized by Garcia, Park, Novelli, and Wright (1999), as long as there is an empty slot adjacent to the enlarged crypt.

A stochastic birth–death process governs the scheduling of division and cell loss events.

Fitness mutations affect in a multiplicative fashion the rate parameters of the birth–death process.

There is a single mutator phenotype that requires only a single mutator mutation. Additional mutator mutations have no effect on the mutation rate.

The loss of the first allele of the TSG has no effect on stem cell fitness.

## 3 RESULTS

### 3.1 TSG inactivation depends on the emergence of a mutator

**FIGURE 1:**
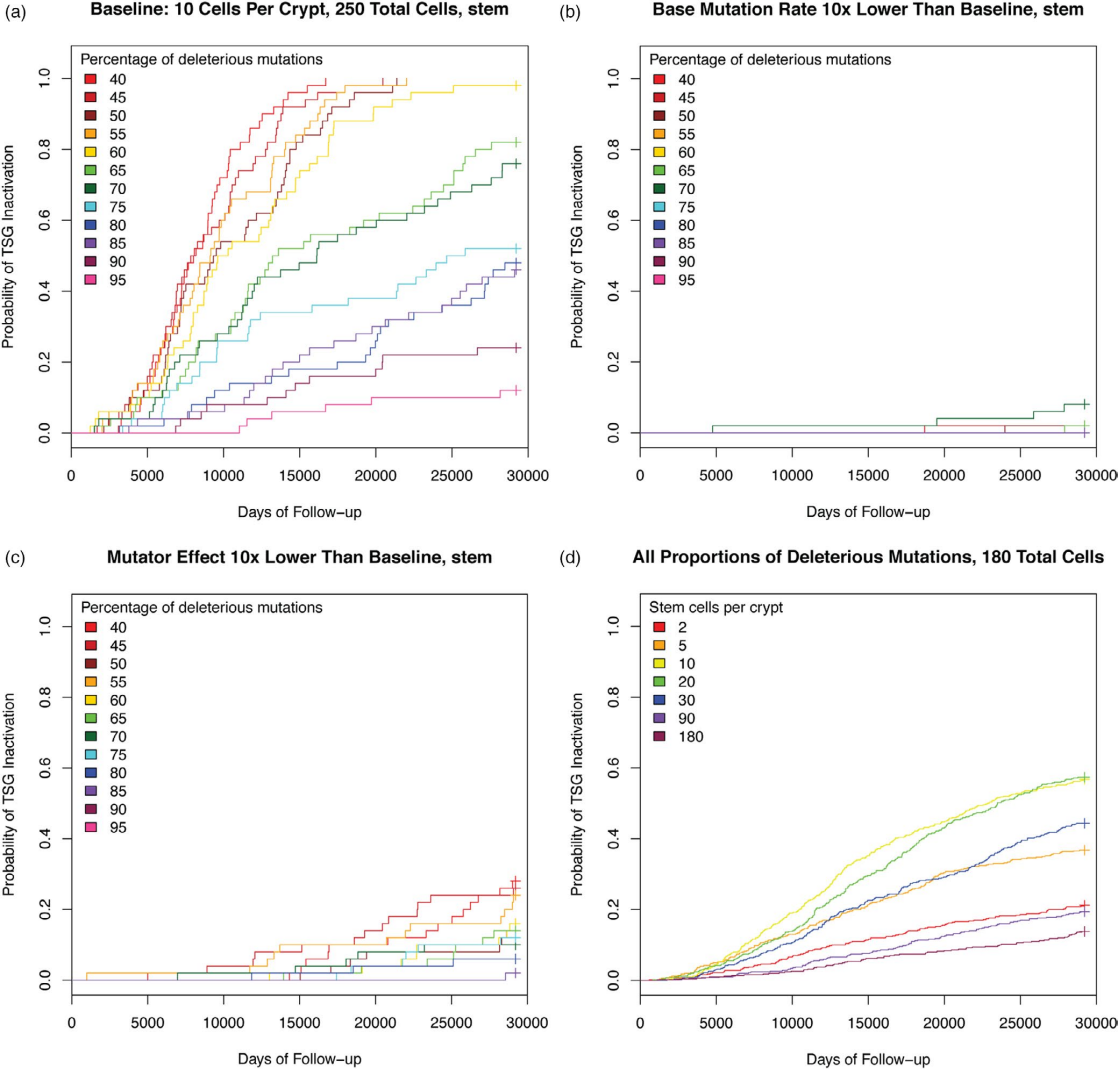
Plots of cumulative hazard functions using the Kaplan–Meier estimator. The tissue was 5 x 5 crypts with 10 stem cells per crypt. In panels (a) through (d), each colored line represents the function for a specific proportion of deleterious mutations. (a) Baseline experiment with default parameter values (Table 1). (b) Mutation rate reduced to 0.1X of baseline. (c) Mutator phenotype reduced to 0.1X of baseline. (d) Each colored line represents a different number of cells per crypt. All proportions of deleterious mutations were included

At baseline, for comparison, our tissue was a 5x5 hexagonal lattice of crypts, each crypt having 10 stem cells. Stem cell loss and symmetric division rates were balanced. Mutations were acquired stochastically with probabilities defined by proportions starting with 50% deleterious mutations, 40% beneficial mutations, and 10% mutator mutations and ranging in increments to 95% deleterious, 4% beneficial, and 1% mutator (4:1 beneficial versus mutator). The incidence of TSG inactivation decreased as the proportion of deleterious mutations increased (Figure 1a, Table 1).

**TABLE 1:** Baseline simulation parameters

Simulation	
Maximum simulation duration in days	29,220 (80 years)
Stem cell	
Division rate ($r_{\text{base\textbackslash{}\_division}}$)	0.05
Ratio of asymmetric divisions to symmetric divisions	20
Stem cell loss rate ($r_{\text{base\textbackslash{}\_cell\textbackslash{}\_los}s}$)	0.04878
Mutation rate per stem cell division	5 x 10–4
Mutation rate maximum	5 x 10–2
Tumor suppressor gene mutation rate	5 x 10–7 (also tested 5 x 10–8)
Tumor suppressor gene mutation rate maximum	5 x 10–5
Number of transient amplifying cells associated with each stem cell	2048
Cell division minimum time in days	0.05
Cell loss minimum time in days	0
Crypt	
Target equilibrium level of number of stem cells in a crypt ($k_{\text{crypt\textbackslash{}\_size}}$)	default 10 (varied from 2–180)
Standard deviation from equilibrium level. the crypt uses this value to determine its level of effect on the cell loss and division rates of the stem cells ($k_{\text{crypt\textbackslash{}\_deviation}}$)	2.0
Bifurcation threshold factor below which a crypt will not bifurcate	2.0
Crypt cell loss effect multiplier	2.0
Crypt division effect multiplier	2.0
Multiplier to the division effect for each dead neighbor crypt	2
Cancer	
Uncontrolled cell proliferation threshold. If a crypt has this threshold times the equilibrium number of stem cells, it is considered to be experiencing uncontrolled stem cell.	4
Number of tumor suppressor gene hits that mean cancer	2
Mutation	
Percent of non‐neutral mutations that are deleterious	45%–95%
The factor affecting the loss rate of the stem cell from a beneficial mutation ($1/k_{\text{fitness}}$)	0.990099
The factor affecting the division rate of the stem cell from a beneficial mutation ($k_{\text{fitness}}$)	1.01
The factor affecting the cell loss rate of the stem cell from a deleterious mutation ($k_{\text{fitness}}$)	1.01
The factor affecting the division rate of the stem cell from a deleterious mutation ($1/k_{\text{fitness}}$)	0.990099
The factor affecting the mutation rate of the stem cell from a mutator mutation	100 (also tested 10)

Reducing the base mutation rate to 10% of baseline, we observed a marked decrease in TSG inactivation (Figure 1b), as expected. Similarly, reducing the effect of the mutator mutation to 10X the baseline mutation rate, instead of 100X, produced a significant decrease in TSG inactivation (Figure 1c). We found that the vast majority of TSG inactivation occurred in stem cells that had previously acquired the mutator phenotype (Figure S2). This assumes that the mutator phenotype can be caused by a single mutation that is otherwise neutral (e.g., overexpression of DNA polymerase beta (Canitrot et al., 1998) or a dominant‐negative mutation in p53 (de Vries et al., 2002), though this assumption is easily relaxed.

### 3.2 Proportion of deleterious mutations negatively correlates with TSG inactivation

**FIGURE 2:**
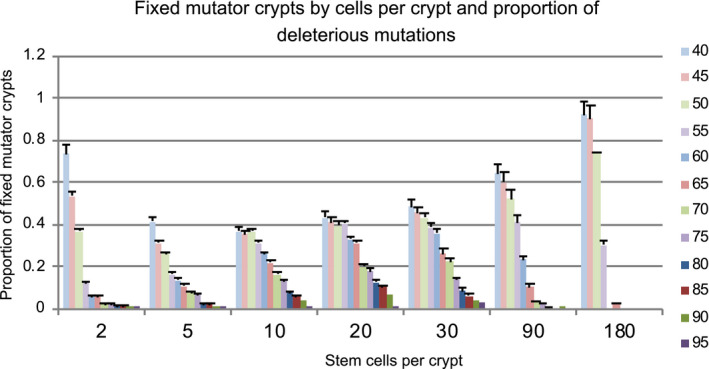
This graph represents the proportion of crypts at the end of the run that contained a population of stem cells with the mutator mutation fixed. Each bar corresponds to a specific proportion of deleterious mutations. The proportion of mutator crypts correlates with the risk of tumor initiation as seen in Figure 1

Not surprisingly, we found that the proportion of deleterious mutations and the incidence of TSG inactivation were negatively correlated (Figure 2). Cell divisions per time remained roughly constant across all proportions of mutations. However, as the proportion of deleterious mutations decreased, the cost of being a mutator also decreased, because it accumulated less mutational burden of deterious mutations. This resulted in an increased emergence of crypts with fixed mutator stem cell populations. Conversely, across all experiments, we observed progressively less TSG inactivation as the proportion of deleterious mutations approached our maximum of 95%.

### 3.3 Increased stem cell turnover initially increased and then decreased TSG inactivation

**FIGURE 3:**
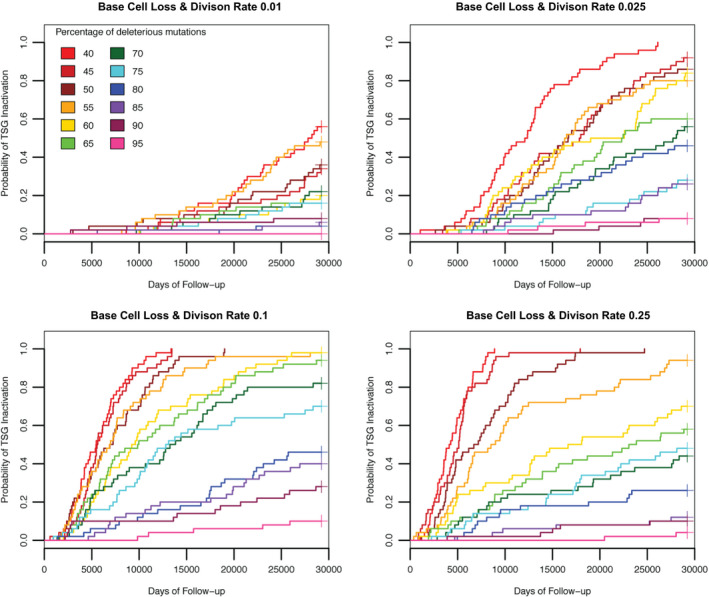
The effect of changes in stem cell turnover. Plots of cumulative hazard functions using the Kaplan–Meier estimator where each colored line represents the function for a specific proportion of deleterious mutations. The baseline division and stem cell loss rates were 0.05 as seen in Figure 1. Initially, turnover correlated positively with the risk of tumor initiation. However, at higher turnover rates, the risk of tumor initiation decreased due to a reduction in the overall number of living stem cells and decreased incidence of mutator crypts

**FIGURE 4:**
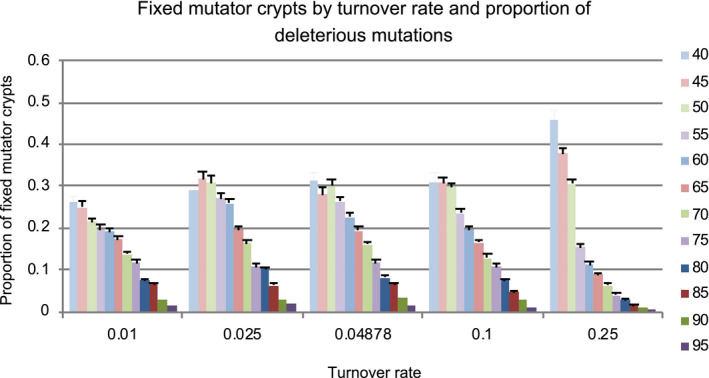
This graph represents the proportion of crypts at the end of the run that contained a population of stem cells with the mutator mutation fixed, as a function of turnover rate. Each bar corresponds to a specific proportion of deleterious mutations. As turnover increased, mutator crypts became more rare at higher proportions of deleterious mutations

We modulated stem cell turnover by varying cell loss and symmetric division rates in unison. At lower turnover levels, we found that increased cell turnover increased TSG inactivation. However, at turnover levels 2x and 5x our baseline level, the incidence of TSG inactivation declined (Figure 3) due to two factors. First, at higher turnover levels, we observed a reduction in the average number of stem cells per crypt (Figure S3). The implemented homeostatic control was unable to maintain the target stem cell population size in the face of high turnover rates. Essentially, there is a lag between depletion of the stem cell pool, due to cell death and differentiation, and replenishment provided by an increase in stem cell division rates. With higher levels of cell loss, the simulated crypts spend more time, further below the target homeostatic number of stem cells. As a result, there were fewer total stem cells in the simulation and therefore fewer mutations per time, allowing less chance for mutator acquisition and TSG inactivation. This may or may not be realistic. Second, as the turnover level increased above our baseline, the number of mutator crypts present in the tissue at any given time decreased (Figure 4). Since increased turnover should lead to increased opportunities for mutator mutations to arise, the decline in mutator crypts was a surprise. However, the loss of mutator crypts is due to intercrypt competition, as described below. Increased turnover led to increased stochastic fluctuations in stem cell numbers and thereby increased crypt extinction events. The reduction in stem cell numbers and mutator crypts combined to produce a reduction in the overall incidence of TSG inactivation as turnover rates increased above baseline. If, in reality, homeostatic control of stem cell numbers prevents increased crypt extinctions with increased stem cell turnover, this result would likely not hold. However, all things being equal, increased cell turnover would be expected to increase stem cell number fluctuations.

### 3.4 The number of stem cells per crypt had a varying effect on TSG inactivation

In general, fewer stem cells per crypt reduced the rate of TSG inactivation, even though the total number of stem cells in the tissue was held constant (Figure 1d). However, there is a trade‐off between TSG inactivation and tissue death at very low stem cells per crypt. With only one stem cell per crypt, there is no opportunity for homeostatic signals within a crypt to compensate for stem cell loss. In our model, tissues with one stem cell per crypt were mostly unviable and died out before TSG inactivation or the predetermined simulation end time.

At 90 and 180 cells per crypt, we observed a reduction in the incidence of TSG inactivation (Figure 1d) at all but the lowest proportions of deleterious mutations, as was predicted by models of the crypt stem cell niche of single crypts (Komarova, 2005; Michor et al., 2003) (Figure S4). As in other experiments, along with a reduction in the incidence of TSG inactivation, the frequency of fixed mutator crypts was reduced as well. This shows that selection against mutator cells increases as the number of stem cells increases above some threshold, 10 in our model (as long as the majority of non‐neutral mutations are deleterious) supporting the conclusions by Michor et al. and Komarova (Komarova, 2005; Michor et al., 2003). When the stem cell populations are large, it is very unlikely that a crypt will go extinct, and so, there is no intercrypt competition. In this case, the metapopulation dynamics are reduced to the single crypt dynamics.

**FIGURE 5:**
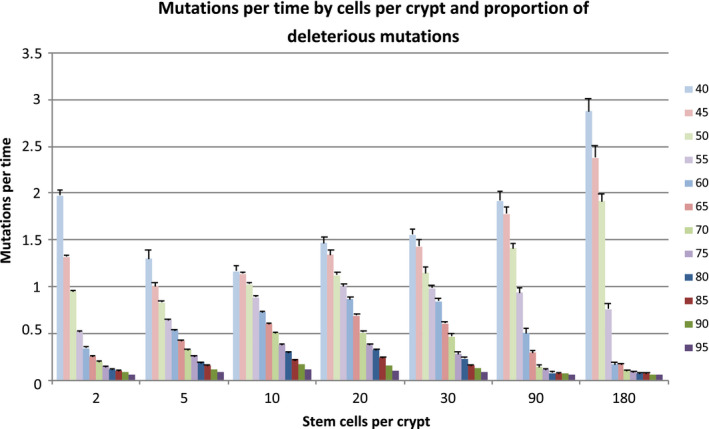
Number of mutations per unit time as a function of cells per crypt and proportion of deleterious mutations where each bar represents a specific proportion of deleterious mutations. At most proportions of deleterious mutations, mutations per time peaked around 20 stem cells per crypt. At 50% deleterious mutations, mutations per time reached a minimum at 5 cells per crypt and increased as the cells per crypt grew past 20

The increased risk of TSG inactivation associated with increased stem cells per crypt appeared to plateau after approximately 10 stem cells per crypt. The total number of cells in the tissue remained constant, as did the total stem cell divisions per time (Figure S5). The average number of mutations per time increased through 10 stem cells per crypt, but then reached a temporary plateau (Figure 5). There was no statistically significant difference between the average number of mutations per time in the 10, 20, and 30 stem cells per crypt cases. As the number of stem cells per crypt increased beyond 30, the average mutations per time decreased except when the proportion of deleterious mutations was 50%. The incidence of mutator crypts followed a similar trend (Figure 2).

We have chosen to explore the case where the total number of stem cells is kept constant, assuming that a certain number of self‐renewing tissue stem cells might be required to maintain an epithelial tissue. An alternative view is that a fixed number of epithelial units, like the crypts, might be required to maintain a tissue and that the number of stem cells per crypt could vary by changing the number of differentiated cells produced by each stem cell. In this case, the risk of TSG inactivation continues to increase with increasing number of stem cells per crypt (Figure S4). Because we held the number of crypts constant but changed the number of cells per crypt in this case, the total number of cells in this simulation also changed, leading to a large evolving population size of stem cells, and thus an increased chance for at least one cell to inactivate both alleles of the TSG.

### 3.5 Partitioning the tissue into crypts imposed a metapopulation dynamic

**FIGURE 6:**
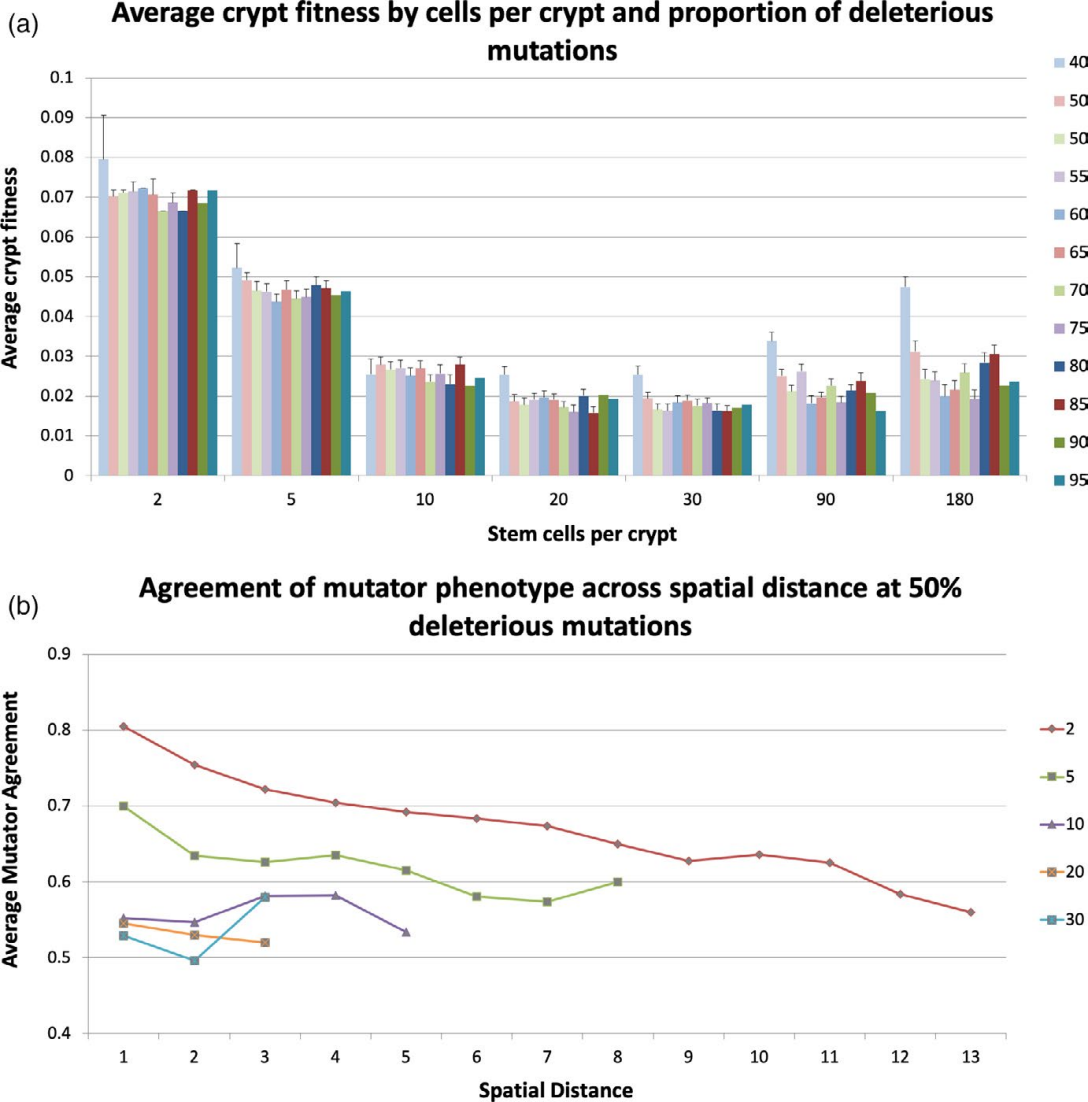
(a) Crypt fitness, measured by the difference between division and cell loss rates, across cells per crypt and proportion of deleterious mutations. The total number of stem cells remained constant across these experiments. The crypt turnover at 2 and 5 cells per crypt allowed fit clones to spread across the tissue resulting in increased overall fitness. (b) Mutator agreement by distance class. Two crypts were in “mutator agreement” if they both had fixed mutator mutations or neither did. Overall agreement was higher in the smaller crypts suggesting that mutator clones were able to spread across the tissue

Fitness levels measured by the difference between division and loss rates were greater in tissues with smaller crypts even as the total number of stem cells remained constant (Figure 6a). Counts of crypt births and crypt life span measurements showed that there was more crypt turnover in smaller crypts (Figures S6 and S7). This crypt turnover provided an opportunity for fitter phenotypes to spread more easily across the tissue. Crypt fitness peaked at 2 cells per crypt where there was the most crypt turnover and bottomed out at 20 and 30 cells per crypt. After 30 cells per crypt, we observed no crypt death and therefore no turnover. Crypt fitness began to increase again at 90 and 180 cells per crypt through natural selection of stem cells within larger crypts. However, fitness levels did not increase to the levels seen at 2 cells per crypt.

The metapopulation dynamic was observed in the clonal expansion of mutator crypts across the tissue. We considered two crypts to have “mutator phenotype agreement” if they both have a fixed mutator mutation or neither has a fixed mutator. We calculated mutator crypt agreement across spatial distance and found that overall agreement decreased as the cells per crypt increased (Figure 6b). Further, at 2 and 5 cells per crypt, closer crypts had increased mutator agreement suggesting clonal expansion of mutator crypts. Conversely, at 10 cells per crypt and above, we found that spatial distance correlated less well with mutator agreement indicating that when there was less crypt turnover, mutator crypts arose de novo rather than through crypt‐level clonal expansion.

## 4 DISCUSSION

In the colon, the development of adenomatous polyps frequently involves the inactivation of the APC gatekeeper gene, a member of the Wnt‐signaling pathway, which represses proliferation and facilitates orderly cell differentiation in the luminal part of the crypts (Barker et al., 2009; Goss & Groden, 2000). As long as this gatekeeper gene is active, mutant stem cell progeny with neoplastic potential is likely eliminated from the crypt and clonal expansion thus averted. However, when the gatekeeper gene is inactivated, the brakes on mutant stem proliferation are removed and mutant cell progeny may undergo focal clonal expansion. Therefore, we asked the question, what are the factors that determine the risk of a TSG/gatekeeper inactivation leading to tumor initiation in a compartmentalized tissue (a metapopulation)?

Our microsimulations indicate that the base mutation rate (Figure 1b) and the increase in that rate for a mutator clone (Figure 1c) are the main driving forces behind TSG inactivation and thus the initiation of carcinogenesis. The proportion of deleterious mutations is important for its effect on selection of the mutator clone. We find that if most mutations are assumed to be deleterious, then a mutator clone quickly accumulates a large genetic load of deleterious mutations and tends to be driven to extinction in competition with nonmutator cells. However, if mutations are more likely to be beneficial, then a mutator clone can spread more easily, which leads to the early inactivation of both alleles of the tumor suppressor gene (Figure 1a). Similarly, increasing turnover of the stem cells effectively increases the mutation rate and consequently the rate of initiation. However, when turnover is very high, the homeostatic feedback signals cannot maintain the target number of stem cells and so the total stem cell population of the epithelium decreases, which in turn decreases the chances for initiation (Figure 3). Since the rate of TSG inactivation is trivially related to the number of stem cells in the system (Figure S8), we have kept the total number of stem cells constant across all our experimental conditions (except for the experiment in Figure S8).

**FIGURE 7:**
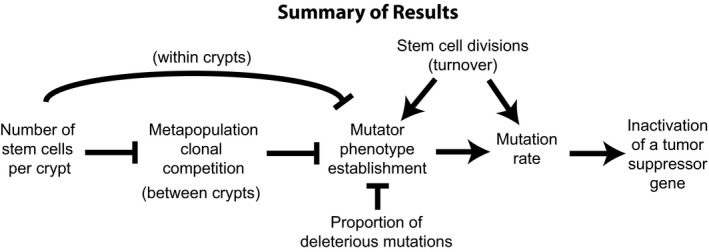
A summary of the results showing the positive and negative effects of the different factors affecting time until both alleles of the tumor suppressor were inactivated

Perhaps the most interesting insight this modeling provides is the importance of the metapopulation dynamics for tumor initiation. When the number of stem cells in a crypt is large enough (e.g., >10 stem cells per crypt), so that there is little crypt turnover, our results match the earlier theoretical findings (Cannataro et al., 2016, 2017; Komarova, 2005; Michor et al., 2003). Mutator clones more readily drift to fixation when there are fewer stem cells and high fitness clones tend to dominate crypts with a large number of stem cells (Figures 4 and 6). Previous work, with a more sophisticated representation of both quiescent and proliferative stem cells, transient amplifying cells, and a distribution of fitness effects of mutations found a balance between suppressing carcinogenesis with fewer stem cells and suppressing the fixation of deleterious alleles and tissue aging with more stem cells per crypt (Cannataro et al., 2016, 2017). However, the story changes dramatically when there are few enough stem cells per crypt that crypts risk extinction and replacement by neighbor crypts. In this case, crypts with fixed mutator clones tend to accumulate more deleterious mutations than nonmutator crypts (Figure 6). Those deleterious mutations tend to lead to more crypt extinctions, and so, the mutator crypts tend to be out‐competed by nonmutator crypts. Since TSG inactivation rarely emerges in a nonmutator crypt, this intercrypt competition actually reduces the chance of initiation. Yet, there is a further countervailing force that prevents the evolution of minimal stem cell populations per crypt. When the stem cell population is so low that crypts die very frequently, crypt bifurcation may not be able to replenish the epithelium quickly enough and the entire epithelium is at risk of extinction. Thus, we predict that there are two optima of stem cell population sizes for crypts as a result of selection to suppress cancer at the level of organisms: small stem cell population sizes that allow intercrypt competition to suppress mutator clones and crypts with very large stem cell sizes that can suppress mutator clones within each crypt due to intracrypt competition. Note that these predictions do not hold if the majority of non‐neutral mutations are beneficial to a clone (see the 50% deleterious mutations in Figures 2 and 5). Recent evidence suggests that this may actually be the case (Martincorena et al., 2017). In that case, a mutator clone would increase in fitness over time, in which case selection at the organismal level should produce crypts with enough stem cells to prevent intercrypt competition, but few enough that genetic drift can limit the spread of selectively advantageous (and mutator) clones. The exact size of crypts optimized for suppressing cancer depends on the details of within‐ and between‐crypt homeostasis, which are unknown. Furthermore, we have assumed that the mutation rate in transient amplifying cells is the same as the mutation rate in stem cells. Previous models have shown that differences in those rates affect the optimal number of stem cells per crypt (Frank et al., 2003; Komarova, 2005) and that cancer may arise from the transient amplifying cell compartment, if it is initiated by mutations that allow those cells to avoid further differentiation (Haeno, Levine, Gilliland, & Michor, 2009; Jilkine & Gutenkunst, 2014). All of the interactions between the different factors governing time until TSG inactivation are summarized in Figure 7.

Our models predict that inactivation of a tumor suppressor gene early in cancer progression tends to occur in cells that are genetically or epigenetically unstable. Unstable clones, however, appear to have difficulty expanding in competition with stable clones, as long as more than 50% of non‐neutral (epi)genetic alterations are deleterious for the clone. Our model also predicts that the selective pressure of cancer has either led to the “architectural” evolution of epithelial proliferative units with large numbers of stem cells and virtually no metapopulation dynamics (Clayton et al., 2007), or small numbers of stem cells within each proliferative unit, with frequent expansions of clones with fitness advantages, as is seen in p53 mutant clones the skin (Chao et al., 2008; Zhang et al., 2005). We predict that proliferative units with moderate‐sized stem cell populations (e.g., 10–20 stem cells per crypt) would have been particularly cancer prone and so selected against in the history of multicellular organisms with self‐renewing proliferative tissues.

This work highlights the importance of crypt (tissue‐level) dynamics. Unfortunately, little is known about the life cycle of crypts (Totafurno et al., 1987) and other epithelial subpopulation structures (Chao et al., 2008; Zhang et al., 2005) that constrain the dynamics of clonal expansions and mutator clones in epithelia. Since the vast majority of cancers arise in epithelia, these dynamics are likely to constrain the evolution of most cancers.

We have assumed that there is some form of homeostatic mechanism for maintaining a relatively constant number of stem cells per crypt. Crypts that are challenged by acute cytotoxic insults such as ionizing radiation (Roberts, Hendry, & Potten, 1995) are known to regenerate from surviving stem cells or clonogenic cells suggesting the presence of homeostatic feedback mechanisms in the crypt. However, in most organs, the mechanism and even presence of a homeostatic mechanism for maintaining stem cell numbers is unknown. This has important implications for carcinogenesis. In the absence of homeostasis, there should be increased metapopulation dynamics and selection between epithelial proliferative units like crypts. In contrast, with very effective homeostatic mechanisms, an epithelial proliferative unit would be unlikely to go to extinction, except in the presence of external insults, and so, the beginnings of carcinogenesis in the intestine would reduce to wound healing dynamics and mutational events solely controlled by intracrypt dynamics. In addition, there has likely been a variety of selective pressures that have shaped crypt biology, including not just tissue maintenance, but also efficiency of nutrient and liquid absorption, regulation of the intestinal microbiome, etc. In the absence of knowledge about homeostasis in crypts, and other, noncancer, selective pressures on crypt dynamics, the results of our model are qualitative, not quantitative.

Another important caveat to our results is the limitation of the size of the simulation. We have represented a small portion of the surface of the colon—just a 5x5 grid of crypts in most cases. To compensate for the small number of cells simulated, we have used unrealistically high mutation rates, though this still might not be sufficient to capture realistic stochastic dynamics (Loeb et al. 2019). This is a further reason to interpret our results qualitatively, but not to extract quantitative predictions of the optimal number of stem cells in a crypt.

For simplicity, we have modeled the mutator phenotype as a result of a single hit, which, if taken literally, can be thought to represent either a dominant‐negative mutation, an increase in expression driven by a single allele, or a haploinsufficient locus. It can also be thought of as the low probability of the second hit in a recessive locus. It would not be difficult to elaborate the model to represent an initial neutral mutation in a mutator locus followed by a second hit that causes the mutator phenotype. The qualitative results should be the same.

Previous theoretical work suggested that deleterious somatic mutations are unlikely to affect the dynamics of carcinogenesis (Beckman & Loeb, 2005; Beckman, 2009). However, in this study we show that the proportion of mutations that are deleterious can have a dramatic effect on the spread of a mutator clone (Figure 2), which in turn dramatically accelerates the process of neoplastic progression (Figure 1). Although our results are not inconsistent with previous findings—a reduction in the ratio of mutator pathways versus nonmutator pathways in the presence of deleterious mutations—there are important differences worth pointing out. For example, the study by Beckman (Beckman, 2009) considered the effect of a mutator phenotype on independent (normal) cell lineages, rather than in the context of a dynamic metapopulation controlled by tissue architecture and homeostatic conditions. Here, we consider the evolutionary dynamics in a crypt‐structured normal tissue involved in the initiation of precursor lesions such as colorectal polyps rather than cancer which requires additional transformational mutations. Thus, the metapopulation dynamics in a field of normal tissue studied here may well represent a plausible mechanism for intestinal field cancerization the importance of which is still unclear (Williams et al. 2018).

Conflicting levels of selection on organisms and potentially on neoplastic cells can lead to unusual results. In organismal evolution, most non‐neutral mutations are thought to be deleterious because species have been selected in their environments for a long period of time and most beneficial mutations have already spread through the population. This dynamic has been replicated in vitro (Elena & Lenski, 2003). However, the basis for this observation in organismal evolution does not hold for somatic evolution. A large proportion of the genome of a multicellular organism may be devoted to coordinating the functions and cooperation of somatic cells, through differentiation, cell cycle control, and apoptosis (Aktipis et al., 2015; Rajagopalan et al., 2003). In fact, a recent study of mutations across cancers found evidence for positive selection of mutations that increase cell level fitness in an order of magnitude more genes (2.2%) than genes with evidence for negative selection of deleterious mutations (0.14%) (Martincorena et al., 2017), though it remains possible that negative selection may be suppressed by deleterious mutations hitchhiking on clonal expansions driven by beneficial mutations and by fixation of weakly deleterious genes in small stem cell compartments due to genetic drift, as we have shown in our model.

Our microsimulations indicate that smaller crypt compartments (with < 10 stem cells) are more efficient in delaying tumor initiation because they are more efficient at purging crypts with deleterious mutations and therefore are more efficient at purging mutator crypts that tend to accumulate deleterious mutations (assuming the majority of non‐neutral mutations are deleterious). This metapopulation dynamic is revealed by crossing scales from stem cell dynamics to crypt‐level dynamics. Our model highlights the importance of answering three key biological questions in order to understand the dynamics of neoplastic progression in epithelial tissues: 1) What proportion of non‐neutral mutations are deleterious for a premalignant clone? 2) What are the homeostatic feedback mechanisms (if any) that regulate the number of stem cells in a proliferative unit like an intestinal crypt? and 3) What are the turnover (and homeostasis) dynamics of epithelial proliferative units? Answering these questions and developing interventions that modulate those dynamics might allow us to control tumor initiation and progression more effectively. This is particularly important for individuals that carry genetic alleles that confer a higher risk of cancer, by abrogating the tissue defense mechanisms against clonal expansions.

## CONFLICTS OF INTEREST

The authors declare no conflicts of interest.

## Supporting information

Fig S1‐S8

## Data Availability

The data that support the findings of this study are openly available: [dataset]David Birtwell, Georg Luebeck and Carlo C. Maley; 2020; Stem‐Sim; Dryad***; Version 0; DOI: *** The source code for the model is freely available: [source code]David Birtwell, Georg Luebeck and Carlo C. Maley; 2020; Stem‐Sim; https://github.com/augustearth/stem-sim; Version 0; https://doi.org/10.5281/zenodo.3809110.

## References

[eva13069-bib-0001] Aktipis, C. A. , Boddy, A. M. , Jansen, G. , Hibner, U. , Hochberg, M. E. , Maley, C. C. , & Wilkinson, G. S. (2015). Cancer across the tree of life: Cooperation and cheating in multicellularity. Philosophical Transactions of the Royal Society of London Series B, Biological Sciences, 370(1673), 20140219 10.1098/rstb.2014.0219 26056363 PMC4581024

[eva13069-bib-0002] Barker, N. , Ridgway, R. A. , van Es, J. H. , van de Wetering, M. , Begthel, H. , van den Born, M. , … Clevers, H. (2009). Crypt stem cells as the cells‐of‐origin of intestinal cancer. Nature, 457(7229), 608–611. 19092804 10.1038/nature07602

[eva13069-bib-0003] Beckman, R. A. (2009). Mutator mutations enhance tumorigenic efficiency across fitness landscapes. PLoS One, 4(6), e5860 10.1371/journal.pone.0005860 19517009 PMC2690659

[eva13069-bib-0004] Beckman, R. A. , & Loeb, L. A. (2005). Negative clonal selection in tumor evolution. Genetics, 171(4), 2123–2131. 10.1534/genetics.105.040840 16143627 PMC1456124

[eva13069-bib-0005] Bielas, J. H. , Loeb, K. R. , Rubin, B. P. , True, L. D. , & Loeb, L. A. (2006). Human cancers express a mutator phenotype. Proceedings of the National Academy of Sciences of the United States of America, 103(48), 18238–18242. 10.1073/pnas.0607057103 17108085 PMC1636340

[eva13069-bib-0006] Breivik, J. (2005). The evolutionary origin of genetic instability in cancer development. Seminars in Cancer Biology, 15(1), 51–60. 10.1016/j.semcancer.2004.09.008 15613288

[eva13069-bib-0007] Cairns, J. (1975). Mutation selection and the natural history of cancer. Nature, 255, 197–200. 10.1038/255197a0 1143315

[eva13069-bib-0008] Canitrot, Y. , Cazaux, C. , Frechet, M. , Bouayadi, K. , Lesca, C. , Salles, B. , & Hoffmann, J. S. (1998). Overexpression of DNA polymerase beta in cell results in a mutator phenotype and a decreased sensitivity to anticancer drugs. Proceedings of the National Academy of Sciences of the USA, 95(21), 12586–12590. 9770529 10.1073/pnas.95.21.12586 PMC22874

[eva13069-bib-0009] Cannataro, V. L. , Gaffney, S. G. , & Townsend, J. P. (2018). Effect sizes of somatic mutations in cancer. Journal of the National Cancer Institute, 110(11), 1171–1177. 10.1093/jnci/djy168 30365005 PMC6235682

[eva13069-bib-0010] Cannataro, V. L. , McKinley, S. A. , & St Mary, C. M. (2016). The implications of small stem cell niche sizes and the distribution of fitness effects of new mutations in aging and tumorigenesis. Evolutionary Applications, 9(4), 565–582. 10.1111/eva.12361 27099622 PMC4831459

[eva13069-bib-0011] Cannataro, V. L. , McKinley, S. A. , & St Mary, C. M. (2017). The evolutionary trade‐off between stem cell niche size, aging, and tumorigenesis. Evolutionary Applications, 10(6), 590–602. 28616066 10.1111/eva.12476 PMC5469181

[eva13069-bib-0012] Chao, D. L. , Eck, J. T. , Brash, D. E. , Maley, C. C. , & Luebeck, E. G. (2008). Preneoplastic lesion growth driven by the death of adjacent normal stem cells. Proceedings of the National Academy of Sciences of the United States of America, 105(39), 15034–15039. 10.1073/pnas.0802211105 18815380 PMC2567488

[eva13069-bib-0013] Clayton, E. , Doupe, D. P. , Klein, A. M. , Winton, D. J. , Simons, B. D. , & Jones, P. H. (2007). A single type of progenitor cell maintains normal epidermis. Nature, 446(7132), 185–189. 17330052 10.1038/nature05574

[eva13069-bib-0015] de Vries, A. , Flores, E. R. , Miranda, B. , Hsieh, H. M. , van Oostrom, C. T. , Sage, J. , & Jacks, T. (2002). Targeted point mutations of p53 lead to dominant‐negative inhibition of wild‐type p53 function. Proceedings of the National Academy of Sciences of the United States of America, 99(5), 2948–2953. 10.1073/pnas.052713099 11867759 PMC122453

[eva13069-bib-0016] Elena, S. F. , & Lenski, R. E. (2003). Evolution experiments with microorganisms: The dynamics and genetic bases of adaptation. Nature Reviews Genetics, 4(6), 457–469. 10.1038/nrg1088 12776215

[eva13069-bib-0017] Frank, S. A. , Iwasa, Y. , & Nowak, M. A. (2003). Patterns of cell division and the risk of cancer. Genetics, 163(4), 1527–1532. 12702695 10.1093/genetics/163.4.1527 PMC1462514

[eva13069-bib-0018] Garcia, S. B. , Park, H. S. , Novelli, M. , & Wright, N. A. (1999). Field cancerization, clonality, and epithelial stem cells: the spread of mutated clones in epithelial sheets. The Journal of Pathology, 187, 61–81. 10.1002/(SICI)1096-9896(199901)187:1<61:AID-PATH247>3.0.CO;2-I 10341707

[eva13069-bib-0019] Goss, K. H. , & Groden, J. (2000). Biology of the adenomatous polyposis coli tumor suppressor. Journal of Clinical Oncology: Official Journal of the American Society of Clinical Oncology, 18(9), 1967–1979. 10.1200/JCO.2000.18.9.1967 10784639

[eva13069-bib-0020] Haeno, H. , Levine, R. L. , Gilliland, D. G. , & Michor, F. (2009). A progenitor cell origin of myeloid malignancies. Proceedings of the National Academy of Sciences of the United States of America, 106(39), 16616–16621. 10.1073/pnas.0908107106 19805346 PMC2757818

[eva13069-bib-0021] Hanski, I. , & Gaggiotti, O. E. (2004). Ecology, genetics, and evolution of metapopulations. London, UK: Elsevier Academic Press.

[eva13069-bib-0022] Herr, A. J. , Kennedy, S. R. , Knowels, G. M. , Schultz, E. M. , & Preston, B. D. (2014). DNA replication error‐induced extinction of diploid yeast. Genetics, 196(3), 677–691. 10.1534/genetics.113.160960 24388879 PMC3948800

[eva13069-bib-0023] Humphries, A. , & Wright, N. A. (2008). Colonic crypt organization and tumorigenesis. Nature Reviews Cancer, 8(6), 415–424. 10.1038/nrc2392 18480839

[eva13069-bib-0024] Ji, H. P. , & King, M. C. (2001). A functional assay for mutations in tumor suppressor genes caused by mismatch repair deficiency. Human Molecular Genetics, 10(24), 2737–2743. 10.1093/hmg/10.24.2737 11734538

[eva13069-bib-0025] Jilkine, A. , & Gutenkunst, R. N. (2014). Effect of dedifferentiation on time to mutation acquisition in stem cell‐driven cancers. PLoS Computational Biology, 10(3), e1003481 10.1371/journal.pcbi.1003481 24603301 PMC3945168

[eva13069-bib-0026] Komarova, N. L. (2005). Cancer, aging and the optimal tissue design. Seminars in Cancer Biology, 15(6), 494–505. 10.1016/j.semcancer.2005.07.003 16143543

[eva13069-bib-0027] Komarova, N. L. , & Cheng, P. (2006). Epithelial tissue architecture protects against cancer. Mathematical Biosciences, 200(1), 90–117. 10.1016/j.mbs.2005.12.001 16427657

[eva13069-bib-0028] Kostadinov, R. , Maley, C. C. , & Kuhner, M. K. (2016). Bulk genotyping of biopsies can create spurious evidence for hetereogeneity in mutation content. PLoS Computational Biology, 12(4), e1004413 10.1371/journal.pcbi.1004413 27105344 PMC4841575

[eva13069-bib-0029] Loeffler, M. , Birke, A. , Winton, D. , & Potten, C. (1993). Somatic mutation, monoclonality and stochastic models of stem cell organization in the intestinal crypt. Journal of Theoretical Biology, 160(4), 471–491. 10.1006/jtbi.1993.1031 8501919

[eva13069-bib-0030] Loeffler, M. , Bratke, T. , Paulus, U. , Li, Y. Q. , & Potten, C. S. (1997). Clonality and life cycles of intestinal crypts explained by a state dependent stochastic model of epithelial stem cell organization. Journal of Theoretical Biology, 186(1), 41–54. 10.1006/jtbi.1996.0340 9176636

[eva13069-bib-0031] Maley, C. C. , Galipeau, P. C. , Li, X. , Sanchez, C. A. , Paulson, T. G. , Blount, P. L. , & Reid, B. J. (2004). The combination of genetic instability and clonal expansion predicts progression to esophageal adenocarcinoma. Cancer Research, 64(20), 7629–7633. 10.1158/0008-5472.CAN-04-1738 15492292

[eva13069-bib-0032] Maley, C. C. , Galipeau, P. C. , Li, X. , Sanchez, C. A. , Paulson, T. G. , & Reid, B. J. (2004). Selectively advantageous mutations and hitchhikers in neoplasms: p16 lesions are selected in Barrett’s esophagus. Cancer Research, 64(10), 3414–3427. 10.1158/0008-5472.CAN-03-3249 15150093

[eva13069-bib-0033] Martincorena, I. , Raine, K. M. , Gerstung, M. , Dawson, K. J. , Haase, K. , Van Loo, P. , … Campbell, P. J. (2017). Universal patterns of selection in cancer and somatic tissues. Cell, 171(5), 1029–1041. 10.1016/j.cell.2017.09.042 29056346 PMC5720395

[eva13069-bib-0034] Martinez, P. , Mallo, D. , Paulson, T. G. , Li, X. , Sanchez, C. A. , Reid, B. J. , … Maley, C. C. (2018). Evolution of Barrett’s esophagus through space and time at single‐crypt and whole‐biopsy levels. Nature Communications, 9(1), 794 10.1038/s41467-017-02621-x PMC5824808 29476056

[eva13069-bib-0035] Meineke, F. A. , Potten, C. S. , & Loeffler, M. (2001). Cell migration and organization in the intestinal crypt using a lattice‐free model. Cell Proliferation, 34(4), 253–266. 10.1046/j.0960-7722.2001.00216.x 11529883 PMC6495866

[eva13069-bib-0036] Michor, F. , Frank, S. A. , May, R. M. , Iwasa, Y. , & Nowak, M. A. (2003). Somatic selection for and against cancer. Journal of Theoretical Biology, 225(3), 377–382. 10.1016/S0022-5193(03)00267-4 14604590

[eva13069-bib-0037] Nowak, M. A. , Michor, F. , & Iwasa, Y. (2003). The linear process of somatic evolution. Proceedings of the National Academy of Sciences USA, 100(25), 14966–14969. 10.1073/pnas.2535419100. PMC299861 14657359

[eva13069-bib-0038] Pepper, J. W. , Findlay, S. C. , Kassen, R. , Spencer, S. L. , & Maley, C. C. (2009). Cancer research meets evolutionary biology. Evolutionary Applications, 2(1), 62–70. 25567847 10.1111/j.1752-4571.2008.00063.x PMC3352411

[eva13069-bib-0039] Pepper, J. W. , Sprouffske, K. , & Maley, C. C. (2007). Animal cell differentiation patterns suppress somatic evolution. PLoS Computational Biology, 3(12), e250 10.1371/journal.pcbi.0030250 18085819 PMC2134960

[eva13069-bib-0040] Rajagopalan, H. , Nowak, M. A. , Vogelstein, B. , & Lengauer, C. (2003). The significance of unstable chromosomes in colorectal cancer. Nature Reviews Cancer, 3, 695–701. 10.1038/nrc1165 12951588

[eva13069-bib-0041] Roberts, S. A. , Hendry, J. H. , & Potten, C. S. (1995). Deduction of the clonogen content of intestinal crypts: A direct comparison of two‐dose and multiple‐dose methodologies. Radiation Research, 141(3), 303–308. 10.2307/3579007 7871157

[eva13069-bib-0042] Salk, J. J. , Salipante, S. J. , Risques, R. A. , Crispin, D. A. , Li, L. , Bronner, M. P. , Brentnall, T. A. , Rabinovitch, P. S. , Horwitz, M. S. , & Loeb, L. A. (2009). Clonal expansions in ulcerative colitis identify patients with neoplasia. Proceedings of the National Academy of Sciences USA, 106(49), 20871–20876. 10.1073/pnas.0909428106. PMC2779829 19926851

[eva13069-bib-0043] Totafurno, J. , Bjerknes, M. , & Cheng, H. (1987). The crypt cycle. Crypt and villus production in the adult intestinal epithelium. Biophysical Journal, 52(2), 279–294. 3663832 10.1016/S0006-3495(87)83215-0 PMC1330079

[eva13069-bib-0044] Vermeulen, L. , Morrissey, E. , van der Heijden, M. , Nicholson, A. M. , Sottoriva, A. , Buczacki, S. , … Winton, D. J. (2013). Defining stem cell dynamics in models of intestinal tumor initiation. Science, 342(6161), 995–998. 24264992 10.1126/science.1243148

[eva13069-bib-0045] Weisenberger, D. J. , Siegmund, K. D. , Campan, M. , Young, J. , Long, T. I. , Faasse, M. A. , … Laird, P. W. (2006). CpG island methylator phenotype underlies sporadic microsatellite instability and is tightly associated with BRAF mutation in colorectal cancer. Nature Genetics, 38(7), 787–793. 10.1038/ng1834 16804544

[eva13069-bib-0046] Williams, M. J. , Werner, B. , Heide, T. , Curtis, C. , Barnes, C. P. , Sottoriva, A. , & Graham, T. A. (2018). Quantification of subclonal selection in cancer from bulk sequencing data. Nature Genetics, 50(6), 895–903. 10.1038/s41588-018-0128-6 29808029 PMC6475346

[eva13069-bib-0047] Zhang, W. , Hanks, A. N. , Boucher, K. , Florell, S. R. , Allen, S. M. , Alexander, A. , … Grossman, D. (2005). UVB‐induced apoptosis drives clonal expansion during skin tumor development. Carcinogenesis, 26(1), 249–257. 10.1093/carcin/bgh300 15498793 PMC2292404

