# Supplementary material for: The evolution of metapopulation dynamics and the number of stem cells in intestinal crypts and other tissue structures in multicellular bodies
Source: Evol Appl. 2020 Aug 12;13(7):1771–83. doi: 10.1111/eva.13069 (PMC7428809; doi:10.1111/eva.13069)
Supplement: Supplementary file 1 — Fig S1‐S8 [file EVA-13-1771-s001.docx]

**Supplementary Information**

The Evolution of Metapopulation Dynamics and the Number of Stem Cells in Intestinal Crypts and Other Tissue Structures in Multicellular Bodies

Supplementary Figure 1. Basic model flow chart.

Supplementary Figure 2. Almost all cases of dual inactivations occurred in cells that had acquired the mutator phenotype.

Supplementary Figure 3. At higher turnover levels, there is a reduction in the average number of stem cells per crypt.

Supplementary Figure 4. Kaplan-Meier curves showing the time until TSG inactivation given different numbers of stem cells per crypt. Within each panel, the curves show different proportions of deleterious mutations.

Supplementary Figure 5. The number of stem cell divisions per time as function of the number of stem cells per crypt and the percent deleterious mutations (shades of gray). The total number of stem cells in the simulations were held constant across all conditions.

Supplementary Figure 6. The number of crypt divisions (births) as a function of the number of stem cells per crypt and the percent deleterious mutations.

Supplementary Figure 7. The number of days crypts survived as a function of the number of stem cells per crypt and the percent deleterious mutations.

Supplementary Figure 8. Kaplan-Meier curves of the time until TSG inactivation for crypts with different number of stem cells, keeping the total number of crypts constant (a 5x5 hexagonal grid of 25 crypts).
